# Gastroesophageal Reflux Disease: Medical or Surgical Treatment?

**DOI:** 10.1155/2009/371580

**Published:** 2009-12-31

**Authors:** Theodore Liakakos, George Karamanolis, Paul Patapis, Evangelos P. Misiakos

**Affiliations:** ^1^Department of Surgery, University of Athens School of Medicine, Attikon University Hospital, Rimini 1, Chaidari, Athens 124 62, Greece; ^2^Department of Internal Medicine, Gastroenterology Unit, University of Athens School of Medicine, Attikon University Hospital, Rimini 1, Chaidari, Athens 124 62, Greece

## Abstract

*Background*. Gastroesophageal reflux disease is a common condition with increasing prevalence worldwide. The disease encompasses a broad spectrum of clinical symptoms and disorders from simple heartburn without esophagitis to erosive esophagitis with severe complications, such as esophageal strictures and intestinal metaplasia. Diagnosis is based mainly on ambulatory esophageal pH testing and endoscopy. There has been a long-standing debate about the best treatment approach for this troublesome disease. *Methods and Results*. Medical treatment with PPIs has an excellent efficacy in reversing the symptoms of GERD, but they should be taken for life, and long-term side effects do exist. However, patients who desire a permanent cure and have severe complications or cannot tolerate long-term treatment with PPIs are candidates for surgical treatment. Laparoscopic antireflux surgery achieves a significant symptom control, increased patient satisfaction, and complete withdrawal of antireflux medications, in the majority of patients. *Conclusion*. Surgical treatment should be reserved mainly for young patients seeking permanent results. However, the choice of the treatment schedule should be individualized for every patient. It is up to the patient, the physician and the surgeon to decide the best treatment option for individual cases.

## 1. Introduction

Gastroesophageal reflux disease (GERD), recognized as a clinical entity only in the mid-1930s, is now the most common upper gastrointestinal disease in the Western countries, with 10%–20% of the population experiencing weekly symptoms [1–4]. Its prevalence is also increasing in the Far East (Japan) and other areas in Asia [5, 6]. This may be related to increased fat consumption in the diet, and the expanding proportion of obese individuals [7, 8]. 

The disease is characterized by a broad spectrum of clinical symptoms and disorders [9]. According to the Montreal definition and classification of the disease [10], GERD is a condition which develops when the reflux of stomach contents causes troublesome symptoms and/or complications. The disease encompasses esophageal and extra-esophageal syndromes. The esophageal syndromes include the symptomatic syndromes, that is, *the typical reflux syndrome* and *the reflux chest pain syndrome*, and the syndromes with esophageal injury, that is, *reflux esophagitis*, *reflux stricture*, *Barrett's esophagus (BE)* and *esophageal adenocarcinoma*. The extra-esophageal syndromes are respiratory conditions, such as chronic coughing, asthma, laryngitis, otitis media, mainly caused by the reflux of gastric juice into the respiratory tract [11].

GERD is a chronic disease characterized mainly by symptoms of heartburn and acid regurgitation during daily activities. In addition approximately 45% of the symptomatic GERD sufferers have nighttime symptoms (NTG), and patients with NTG have significantly greater odds of having moderate or severe GERD [12]. The aim of therapy for patients with GERD is to achieve symptomatic relief, prevention of relapses and healing in patients with severe esophagitis or complicated disease. These goals can now be achieved with medication, such as proton-pump inhibitors (PPI), which are now the mainstay of medical treatment of GERD. On the other hand, antireflux surgery, open or laparoscopic, has been effectively used for long-term control of the disease [13].

In this review we will try to compare the benefits and drawbacks of both medical and surgical treatment and present the main indications for both options in the management of GERD.

## 2. Natural History

The natural history of GERD has not been well clarified yet [14]. Natural history studies in GERD are usually retrospective and commonly afflicted with a plethora of shortcomings. Two different concepts have been proposed for the natural history of GERD. The traditional concept approaches the disease as a spectrum, emphasizing the potential progress over time of patients along the spectrum [15, 16]. On the mild end of the spectrum is patients with nonerosive reflux disease (NERD) and on the severe end are patients with complicated GERD (erosive esophagitis, stricture, Barrett's esophagus) [17]. This conceptual framework focused on esophageal mucosal injury as the most significant clinical outcome in GERD. A recent large prospective cohort study confirms this concept, showing that true progression from mild to severe disease and even to BE has occurred over 2 years follow up [17].

In contrast, a new concept indicates that GERD is a categorical disease with three distinct entities: *NERD*, *erosive esophagitis*, and *Barrett's esophagus*. These three phenotypes represent different disorders and movement among them is limited, suggesting that those once determined remain true to form [5, 18, 19]. After discontinuing treatment reflux symptoms tend to recur; however, patients within one of the 3 distinct entities will relapse in the same entity and not to any of the other two. This conceptual framework swifts our focus from mucosal injury to mechanisms leading to symptom generation. A large study with an average of 7.6 years monitoring observed that GERD usually does not progress over the time [20]. According to this report, GERD is a chronic disease but not progressive; reflux symptoms tend to recur but the endoscopic findings do not progress in most patients. After discontinuing treatment, patients within one of the 3 dinstict entities will relapse in the same entity and not to any of the other two. However, other studies confirm that progression of NERD to erosive esophagitis is possible in only 10% of GERD patients. The other patients remain within their respective phenotypic presentations [21].

Patients with severe esophagitis are at especially high risk of developing a stricture. One % per year of these patients develops a stricture, which is usually a direct result of interrupted acid suppressive therapy [20]. Long-standing reflux symptoms are a major risk factor for the development of BE. In these patients prolonged acid and perhaps alkaline injury leads to a significant change of esophageal mucosa from its squamous epithelium to a columnar configuration. Moreover, abnormalities in esophageal peristalsis and gastric dysmotility are other factors which may play a significant role in the pathogenesis of BE. Often failure of symptoms to resolve with acid-reducing medication may be attributed to the presence of duodenal contents in the refluxate causing an unablated injury to the esophagus with its associated motility abnormalities [22]. Patients with BE have an increased risk of esophageal adenocarcinoma; actually the incidence of adenocarcinoma in these patients is 40 times greater than that in the general population. In addition, almost 10% of patients with BE have coexistent adenocarcinoma at the time of initial endoscopy [23].

## 3. Pathophysiology

In the lower part of the esophagus, lies a zone of increased pressure, the lower esophageal sphincter (LES), which has as a primary role to prevent reflux of gastric chyme into the esophagus. Factors contributing to this are the intrinsic musculature of the distal esophagus, the sling fibers of the cardia, the crura of the diaphragm, and the intraabdominal pressure [24, 25].

The LES relaxes in response to esophageal peristalsis to allow the passage of bolus into the stomach. Transient lower esophageal sphincter relaxations (TLESRs) are visceral reflexes occurring mainly in response to gastric distension, and their frequency is influenced by foods and smoking. From a therapeutic perspective, GERD is a disorder of both motility and esophageal acid exposure. Motility mechanisms include transient lower esophageal sphincter relaxations (TLESRs), lower esophageal pressure abnormalities (including the presence of hiatal hernia), impaired esophageal peristalsis, and delayed gastric emptying. Among these motility abnormalities, TLESRs are thought to be a leading one. In patients with GERD, TLESRs account for 50%–75% of reflux episodes especially in patients with postprandial or upright reflux [26–29]. In contrast, in patients with mainly supine reflux the LES becomes incompetent and the role of TLESRs decreases [28]. 

According to the classical view of GERD, acid reflux (nadir pH <4) is considered the most important component in the pathogenesis of this disease, based on the induction of heartburn during perfusion of the esophagus with acidic solutions [30]. However, current evidence using the multichannel impedance with pH sensor suggests that weakly acidic events (nadir pH between 4 and 7) in the esophagus are associated with classic GERD symptoms, particularly in patients taking proton pump inhibitors (PPIs) [31, 32]. In addition, studies using bilirubin monitoring together with pH monitoring showed a synergism between acid and bile reflux components in determining esophageal mucosal damage [33, 34]. 

GERD is often associated with a hiatal hernia, especially a *sliding hiatal hernia *(Type I hernia). In this type of hernia the cardia of the stomach is allowed to migrate back and forth between the posterior mediastinum and the peritoneal cavity. Therefore, the gastroesophageal junction is incompetent and large volumes of gastric contents pass unimpeded into the hiatal sac; furthermore the larger the size of the hernia, the greater the risk of abnormal reflux [35, 36]. 

*Helicobacter pylori* (*H. pylori*) infection has also been implicated in the pathogenesis of GERD. *H. pylori *infection may be associated with either increased acid secretion [37] or achlorydria with resultant atrophic gastritis [38], depending on the species of the bacterium and the inflammatory response induced [8]. Observations showing that *H. pylori *negative patients present with more severe esophagitis than H*. pylori *positive ones, suggest that this organism may have a protective role in GERD [39]. Indeed, infection by these bacteria may induce atrophy and a reduction in gastric acid production, resulting in lower risk of development of GERD. In contrast, eradication of *H. pylori* infection may result in a return to normal acid production and exacerbation of GERD [40]. However, more recent clinical research could not find sufficient evidence for a possible role of H. pylori infection in the development of GERD and erosive esophagitis [41]. In clinical practice though, since chronic H. pylori infection is associated with an increased risk for peptic ulceration and gastric cancer, current guidelines recommend H. pylori eradication irrespective of potential effects on GERD [42].

## 4. Diagnostic Methodology

Although many tools are available for diagnosis of GERD, such as endoscopy, manometry, ambulatory pH monitoring, and esophagogram, none of them is considered the gold standard. Herein, the utility and clinical applications of each test will be analyzed.

*Upper gastrointestinal endoscopy* is one of the principal tests used and its main use in patients with GERD is the evaluation of treatment failures and risk management. Endoscopy may detect esophageal mucosal injury due to GERD (erosive esophagitis, ulceration, stricture, Barrett's esophagus). The endoscopists are directed to grade esophageal mucosal breaks with esophagitis according to the Los Angeles Classification of Esophagitis in 1996 [43]. Typical esophagitis is essential for the diagnosis of GERD [43]. The identification of esophagitis is highly specific (90%–95%) for GERD, but the endoscopy has a quit low sensitivity mainly because the majority of symptomatic GERD patients will have no evidence of mucosal injuries [44]. In clinical practice endoscopy is used as a screening test for BE esophagus or esophageal carcinoma in patients with chronic GERD [45, 46]. Thus, all patients ≥50 years of age with 5–10 years of heartburn should perform endoscopic screening for BE and dysplasia. Endoscopy may also play a main role in the concept of “alarm symptoms”, although a recent meta-analysis showed that they performed poorly as diagnostic tests. Proposed alarm symptoms include vomiting, weight loss, dysphagia, anemia, signs of gastrointestinal blood loss, chest pain, or epigastric mass [47]. Among them dysphagia and especially troublesome dysphagia warrants endoscopic evaluation because it can be indicative of a stricture or malignancy. Moreover, in GERD patients with dysphagia without obvious obstructing lesion the potential value of endoscopy with esophageal biopsies increases as eosinophilic esophagitis is recognized as a confounding clinical entity [48]. There is no evidence to support the utility of routine esophageal biopsies in patients with reflux symptoms without dysphagia [49].

In patients with persistent reflux symptoms despite PPI therapy and normal findings on endoscopy a further evaluation is recommended in order to establish the diagnosis of GERD or to identify alternative diagnoses, such as motor esophageal abnormalities (mainly achalasia), functional heartburn, or eosinophilic esophagitis. Thus, *manometry* should be the second diagnostic test in order to evaluate peristaltic function and diagnose achalasia. This test helps to analyze the function of the peristaltic activity of the body of the esophagus and the LES, prior to anti-reflux surgery. Normal pressures at the LES range between 12 and 30 mmHg. A mechanically defective sphincter is defined as having one of the following characteristics: an average resting pressure of less than 6 mmHg, an average length of less than 2 cm or an average length exposed to the positive-pressure environment of the abdominal cavity of less than 1 cm [50]. However, according to the American Gastroenterological Association recommendations [51] manometry is not indicated for confirming a suspected diagnosis of GERD. It is mainly used to establish the diagnosis of dysphagia in cases in which a mechanical obstruction (e.g., stricture) cannot be found. It is also indicated for the preoperative assessment of candidates for antireflux surgery, to exclude achalasia or ineffective peristalsis (<30 mmHg) [52]. In combination with impedance, manometry helps to identify patients with a significant defect in motility disorders, such as achalasia or aperistalsis associated with collagen disease, such as scleroderma [53]. Recent studies showed that high-resolution manometry has a better sensitivity in recognizing atypical pattern of esophageal motor disorders [54]. Moreover, manometry serves to localize the LES for subsequent pH monitoring for documentation of abnormal esophageal acid exposure.

The best method to diagnose acid reflux is the 24-hour *pH test *[2]. Acid reflux episodes are defined as a pH fall <4. An overall score, known as *DeMeester score*, is calculated using a special formula; this value should not exceed 14.7 in normal subjects. Both *catheter and wireless pH monitoring* allow quantification of esophageal acid exposure and assessment of the temporal relationship between symptoms and acid reflux events [55]. Whether the examination should be performed with the patient on or off PPIs is still debated. Testing off-therapy is often recommended for patients with a low index of suspision for reflux disease, to rule out GERD. Thus, in a patient off PPIs with normal pH study, the symptoms under consideration are not attributable to reflux. On-therapy study is usually used while patients are on PPIs twice daily, intending to investigate the hypothesis of residual acid reflux [55, 56]. The threshold acid exposure time for an abnormal pH study done on PPI therapy proposed to be lowered to the level of 1.6% [57]. As the diagnostic yield of on PPIs study is limited [58, 59], inclusion of symptom indices (symptom index, symptom association probability) adds an important dimension to the interpretation of pH monitoring. While the % time pH >4 indicates the presence of abnormal acid, the symptom indices help to identify the causality of a particular symptom with episodes of acid reflux regardless of whether the total esophageal acid exposure is normal or abnormal. A positive pH study on PPIs suggests that patients' persistent heartburn might be related to ongoing acid reflux (presence of abnormal pH suggest insufficient acid inhibition, whereas positive symptom indices with normal pH suggest that heartburn is induced by normal levels of acid exposure) [32, 56, 59, 60]. A negative pH test on PPIs provides convincing evidence that the patients' symptoms are not related to ongoing acid reflux. However, a negative pH test on PPIs does not exclude the possibility of underlying reflux that may be a cofactor in patient's symptom and is being adequately suppressed on PPIs. As the poor tolerability of pH probes could result in a significant decrease in reflux provoking activities [61], the use of wireless pH capsule, that allows combined testing both off and on PPIs, has been suggested to improve the sensitivity of the pH test [55, 62].

In GERD patients who failed PPI twice daily, the use of *esophageal impedance-pH monitoring* is a very promising technique. Multichannel intraluminal impedance monitoring with pH sensor (MII-pH) can detect all types of reflux (acidic, weakly acidic, and weakly alkaline). This test measures the resistance of electrical conductivity of the esophageal content, thus detecting any change of the esophageal pH due to the presence of liquid or gas reflux [63, 64]. Moreover, with the inclusions of several channels, it can detect the direction of bolus movement, thus allowing identification of swallows (anterograde) and reflux events (retrograde) [63]. Therefore, it is superior to pH monitoring in the detection of reflux symptoms associated with weakly acidic or nonacid reflux in patients on PPI therapy [60, 65]. The test can also provide information regarding the height of the reflux column inside the esophagus and the association between symptoms and reflux episodes (using symptom index or symptom association probability) [63]. Nevertheless, the use of an impedance catheter with gastric pH sensor can be used to evaluate whether the gastric acid secretion is sufficiently suppressed by the medication. Thus the most important clinical indication of MII-pH monitoring is the evaluation of patients with persistent symptoms despite PPI therapy [66, 67]. Indeed Mainie et al. [65] and Zerbib et al. [32] showed that in almost 50% of patients on therapy with PPIs twice daily, esophageal symptoms during 24-hour combined MII-pH monitoring were associated with persistent reflux. Although impedance-pH monitoring is the most sensitive technique for detecting all forms of gastroesophageal reflux, the usefulness of the test in the clinical practice has to be addressed. Thus, it is still unclear whether impedance-pH monitoring should be performed on or off PPIs [68]. Moreover, analysis of impedance-pH monitoring is based on symptom associations which have limitations such as sharp cut points, multiple types (symptom index, symptom association probability) and uncertain time windows for analysis [69]. In addition, manual correction of reflux events, which is time consuming, should be performed as the available software tends to overestimate the number of reflux episodes [70].

The chemical composition of the refluxate could be evaluated by using *Bilitec *which assess bile reflux with bilirubin as the surrogate marker. Detection of bilirubin in the refluxate is indicative of duodenogastroesophageal reflux (DGER). A recent study showed that a significant number of persistent symptoms occurred in association with bile reflux as measured by Bilitec [71]. However, the limited availability of the Bilitec and the dietary restrictions that patients should follow during the test, make its future quite obscure. 

Barium esophagogram should be considered primarily in GERD patients that present with dysphagia [52]. The external anatomy of the esophagus and the proximal stomach can be visualized with an *esophagogram*. This can also show the type and size of an associated hiatal hernia. This diagnostic tool is reasonably accurate in cases of severe (98.7%) or moderate (86%) esophagitis, but it has a very low accuracy with mild esophagitis (24.6%) [72–74]. Moreover, reflux of barium during radiographic examination is only positive in 25%–75% of symptomatic patients and is falsely positive in almost 20% of normal controls [75].

The aforementioned diagnostic tests are mostly invasive, costly and usually not readily available for the practicing physicians. The proton-pump inhibitor (PPI) test is an alternative noninvasive test for the diagnosis of GERD at the disposal of every primary care physician. This test comprises a short course of high-dose PPI (usually omeprazole) and is used as a first-line diagnostic strategy [52]. The notion of the test is based on the hypothesis that if a patient reports symptoms consistent with GERD and responds to therapy with PPI, then he/she should have GERD. The strategy of PPI test is not as robust at patients with non-erosive reflux disease as it is in patients with esophagitis. Indeed, a recent clinical review confirms that the clinical need for the PPI test increases as the true prevalence of GERD in esophageal (erosive, non-erosive reflux disease, non-cardiac chest pain) and extraesophageal syndromes decreases. Recently, the Rome III committee suggested that lack of response to full course of PPI is mandatory for the diagnosis of functional heartburn [76]. They also recognized that patients with a normal PPI test and endoscopy, but who respond to PPI treatment should be considered as having GERD.

A limitation of the test is that in a case of a negative response the diagnostic utility of endoscopy is limited as that the putative presence of mucosal injury, which is highly specific for GERD, will likely be healed [49]. In addition, none of the studies so far has determined if a positive response to the PPI test may predict a long-term response to medical treatment [52].

## 5. Treatment Approaches

Life style and dietary modifications, together with antacids have long been the first line of treatment. Decreased fat intake, weight loss, cessation of smoking, elevation of the head of the bed, and avoiding recumbency for 3 hours postprandially, seem to decrease acid exposure of the lower esophagus [77]. In addition, the avoidance of certain foods such as coffee [78], chocolate, alcohol, peppermint, onions and garlic, which are known to reduce LES pressure, seems to help [72]. Although there are no randomized trials to test the efficacy of these measures, expert opinion holds that it is reasonable to educate the patient about factors that may precipitate reflux.

Antacids, alginic acid, and over-the-counter acid suppressants are useful in the symptomatic relief of milder forms of GERD [72, 73, 79]. In the past several trials suggested that effective symptom relief was obtained in the majority of patients taking over-the-counter medication [80, 81].

### 5.1. Acid Suppression for GERD

Histamine 2 receptor antagonists (H2RAs) were the acid suppression therapy of choice from the mid-1970s until the introduction of proton pump inhibitors (PPIs) into clinical practice in the late 1980s [82]. Currently several types of H2RAs-cimetidine, ranitidine, famotidine, and nizatidine, are available over-the-counter. These drugs in regular doses can decrease gastric acid especially after a meal. H2RAs have a much longer duration of action than antacids [73]. However, several drawbacks limit the use of H2RAs in the treatment of GERD. They are not efficacious in the healing of severe esophagitis; in addition, maintenance therapy with standard doses of H2RAs cannot prevent relapses [83]. Today they are used for the treatment of milder forms of the disease and for on-demand therapy, especially for nocturnal symptoms [84]. 

The class of PPIs is the most potent type of acid-suppressive therapy. PPIs are substituted benzimidazoles that irreversibly bind the H^+^K^+^—ATPase, the final step in gastric acid secretion [85]. Members of this group include omeprazole, lansoprazole, pantoprazole, rabeprazole, and esomeprazole. The standard doses of each drug type can exert a similar acid inhibitory effect. Omeprazole is the agent with the longest documented safety record, whereas the newest agents, rabeprazole and pantoprazole may interact less than the other two with cytochome P450 metabolism [86]. Several trials have shown the superiority of PPIs over H2RAs in the treatment of reflux esophagitis. Klinkenberg-Knol et al. [87] have documented effective long-term control of GERD with PPIs, showing that long-term therapy with omeprazole in regular doses (20 to 60 mg/d) maintains healing of esophagitis for up to 11 years. PPIs in standard doses control symptoms in more than 80% of cases and heal esophagitis in almost 90% of cases within a period of 4–8 weeks [88]. A recent metanalysis in patients with esophagitis showed that PPIs exhibit a better healing effect and faster symptom relief than histamine receptor antagonists (H2RAs), which are in turn better than placebo [89]. The success rate of esophagitis healing was 83% after 8 weeks of therapy. Moreover, PPIs are effective for maintenance of esophagitis healing and symptom control in patients who respond to an acute course of therapy for a period of 6–12 months [49, 90]. Whether one PPI is superior to the other is a matter of a matter of controversy. Although data suggest differences among various PPIs with respect to healing rates [91], absolute differences in efficacy are very modest and more pronounced in patients with severe esophagitis [89]. However, individual variability in patient response to PPIs varies widely and in patients not responding to one PPI switching to another one is usually recommended [92].

Administartion of PPIs is not as robust at resolving GERD symptoms in patients with negative endoscopy. Only 61% of patients experienced resolution of heartburn with PPIs, which is still superior compared to 40% with H2RAs administration [93, 94]. Thus, response of patients with NERD to a standard dose of PPI is approximately 20%–30% lower than that of patients with erosive esophagitis.

Inadequate symptom response to once-daily PPI therapy affects up to 40% of GERD patients and is the most common issue faced by gastroenterologists [59]. The majority of these patients originate from the group with NERD and functional heartburn. In the setting of PPI failure, experts recommend an escalation to twice-daily dosing of PPIs to improve symptom relief [49]. However, identification of the potential mechanisms for lack to response to PPI should be considered before the above mentioned therapeutic strategy. Putative mechanisms for failure of PPI treatment include compliance, improper dosing time, weakly acidic reflux, DGER, delayed gastric emptying, esophageal hypersensitivity, eosinophilic esophagitis nocturnal reflux, residual acid reflux reduced PPI bioavailability, and psychological comorbidity [56, 95]. Compliance and dosing time should be assessed in all patients prior to ordering any evaluative test. The optimal timing for PPIs administration is 30 minutes prior to a meal. Among the other mechanisms, most attention is focused on weakly acidic reflux or DGER. In the presence of weakly acidic reflux or DGER, esophageal hypersensitivity to low-intensity reflux events is suggested as the underlying mechanism for symptom generation [96, 97]. Thus, it is clear that treatment success depends on identification of the putative mechanism of the PPI failure. In case of residual acid reflux, increasing the PPI dose to twice daily, switching to another PPI or adding H2RA mainly for noncturnal reflux could offer a successful therapy option [98].

#### 5.1.1. New Agents

New agents have recently emerged for the treatment of patients nonrespondent to PPI treatment. To address this clinical issue, research efforts have focused on “reflux inhibition”—that is, inhibition of transient lower esophageal relaxations (TLESRs), the predominant mechanism of GERD. Thus the *γ*-aminobutyric acid (GABA) type B (GABA_B_) receptor has emerged as one of the most promising drug targets through which TLESRs can be modulated [99]. Thus in patients with positive esophageal impedance test for weakly acidic reflux, treatment with baclofen, a GABA agonist, which reduces the rate of TLESRs, should be considered. Due to the extensive side effect profile of the drug, a low initial dose with a step-up strategy is usually suggested. Another therapeutic option of these patients, especially if the main resistant symptom is regurgitation, is antireflux surgery. A recent study confirmed that patients who were refractory to PPIs and had positive SI or SAP on esophageal impedance successfully underwent laparoscopic Nissen fundoplication [100]. In patients with negative esophageal impedance monitoring, visceral pain modulators could be helpful [56, 98]. These agents (tricyclic antidepressants, trazodone, and selective serotonine reuptake inhibitors), used in non-mood-altering doses, offer their visceral analgesic effect by acting at the central nervous system and/or sensory afferents level. Promotility agents (prokinetics) have been used in conjunction with PPIs for the treatment of GERD [101]. However, very few of these agents have been proven useful. Cisapride, a selective agonist of 5-HT4 receptor has been used in the past for the symptomatic treatment of nocturnal reflux, because it could significantly reduce TLESRs during sleep. However, it has been abandoned due to its documented association with fatal arrhythmias [102]. Tegaserod, a new selective 5-HT4 partial agonist is being used for the treatment of irritable bowel syndrome and is under investigation for the treatment of GERD. In a recent study this drug reduced postprandial esophageal acid reflux episodes, although without an apparent effect on lower esophageal sphincter pressure [103].

#### 5.1.2. Maintenance Therapy

GERD is a chronic disorder that often requires long-term maintenance therapy. Regardless of endoscopic status at diagnosis, the majority of GERD patients will experience relapse within six months of cessation of short-term acid suppression therapy. The strongest data for maintenance therapy are for erosive esophagitis. A recent meta-analysis showed that 75% of patients with erosive esophagitis, who receive an acute treatment with PPIs, would relapse after a period of 6–12 months without maintenance therapy [104]. This review provides evidence that PPIs are the most effective therapy (versus H2RAs and versus placebo) at maintaining remission in patients with esophagitis—both in terms of endoscopic inflammation and symptom relief. Although H2RAs are inferior to PPIs there may be a role for them with PPI-intolerant patients.

In patients with NERD there are differert therapeutic options: daily (continuous), intermittent fixed courses, or on-demand therapy. The role of daily maintenance therapy in patients with NERD is less clear. The above-mentioned review showed that PPIs are superior to placebo and H_2_-RAs for controlling symptoms [104]. As the need for maintenance therapy in patients with NERD is driven by the impact of residual symptoms on their quality of life, on-demand therapy (treatment only when symptoms occur) could be a reasonable approach. Indeed, the proportion of GERD patients that do not require a daily dose of acid suppression to maintain symptom control is estimated to be 20%–40% [105]. A systematic review showed that on-demand therapy with currently available PPI appears to be effective in the long-term management of patients with NERD or mild and uninvestigated forms of GERD, but not in patients with erosive esophagitis [106]. Although PPIs are more effective overall, antacids or alginate-antacids were also have a prominent role, as adjuvant, on-demand therapies [107]. This was because both drugs have the ability to provide a rapid and adequate relief from GERD symptoms; they are most effective once heartburn is already present, whereas PPIs are more effective in preventing heartburn [49, 107]. 

A major concern of the long-term PPI therapy is the potential side effects that can afflict patients who have been chronically treated with these drugs. At first there was a potential risk of atrophic gastritis and/or hypergastrinemia induced carcinoid tumors due to hypochlorhydria. However, these risks are slight if even demonstrable in clinical practice settings [108]. Nowadays, new concerns have been identified; the most convincing data link PPI use with an increased risk of *Clostridium difficile* colitis and bacterial gastroenteritis [108, 109]. With respect to the hip fracture, there is an increased risk which seems to be of relatively low but worthwhile magnitude [110]. The putative mechanism for fracture is the decreased calcium absorption due to acid inhibition [111]. It seems a good medical practice to screen and treat the elderly for osteoporosis irrespective of PPI use [49]. To summarize the available risk/benefit data on PPIs, PPIs should be used for appropriate indications and should not be used in higher doses or for longer durations necessary to achieve the desired outcome.

### 5.2. Surgery for GERD

#### 5.2.1. Open Antireflux Surgery

Antireflux surgery has developed only after it was documented in the 1950s that a hiatal hernia was associated with GERD [112]. At the beginning, when hiatal hernia was considered the major factor in the production of GERD, antireflux surgery was performed to reduce the hiatal hernia and keep the LES within the peritoneal cavity [113]. Later, when low LES pressure was considered the major factor in the incompetence of the gastroesophageal junction, antireflux procedures were performed to increase LES pressure [114]. Fundoplication was first introduced by Nissen in 1956, after the incidental observation that a fundal patch used to reinforce the esophageal suture line could also correct gastro-esophageal reflux. In the following years, the Belsey and Toupet [115] (partial wrap, 180–200°) procedure has been applied for the treatment of GERD. Despite this relatively short history, antireflux surgery techniques have been gradually advanced overtime resulting in gradual improvement in the clinical outcome [114, 116]. 

As of today, given that transient LES relaxation (TLESR) is the main mechanism responsible for GERD, the aim of surgery is to lengthen the intraabdominal portion of the LES, to reduce the volume of the gastric fundus and prevent effacing of the LES during distention of the stomach postprandially [117].

Following four decades of treatment with open antireflux procedures, the long-term clinical outcome after open surgery has been well described. DeMeester et al. [13], as well as other authors [118, 119], have reported a 90% long-term reflux control after Nissen fundoplication. 

After total fundic wrap several adverse consequences may occur; these include persistent dysphagia, inability to belch and vomit, epigastric fullness, bloating and pain postprandially, temporary swallowing discomfort, and sometimes intense flatus [120]. Many of these postfundoplication side-effects have been included under the term “*gas bloat syndrome*”. Indeed it has been demonstrated that there is an increase in LOS tone after these procedures more prominent after total fundoplication [121]. The rise in the sphincter pressure occurs when the lower esophagus is placed in the intra-abdominal position, where it is in a positive pressure environment [122]. A possible explanation could be that total fundoplication may overcorrect the mechanical deficiency in the gastroesophageal junction, creating a supercompetent cardia [123]. 

On the other hand several studies have demonstrated that these operations reduce the hiatal hernia and restore the physiology of the gastroesophageal junction to normal. Nissen fundoplication reduces postprandial reflux by affecting the frequency of TLOSR. Furthermore, these operations render swallowing-induced LES relaxation incomplete by compressing mechanically the LES segment [120, 123]. Toupet fundoplication only encircles half of the esophageal circumference and thus the basal LES sphincter tone is significantly lower than in Nissen procedure [121]. This procedure normalizes LES tone, without impairing the ability of the LES to relax on proper stimulation [124]. Moreover, it seems to maintain the same high level of reflux control as Nissen procedure. It has been shown that patients after the Toupet procedure have less troublesome flatus and maintain their ability to belch, without jeopardizing important reflux-preventing mechanisms [120, 125].

In the past it has been supported that patients with poor esophageal motility (distal esophageal body contraction amplitude <30 mmHg) be treated with a partial fundoplication rather than a total one, to avoid the side effect of the latter. However, this idea was not supported by the results of a randomized clinical trial [126]. There are clinical advantages in doing a Toupet procedure, but there are also some technical modifications of the Nissen procedure which help in minimizing these side-effects [127, 128]. The preferred and most efficient modification of the Nissen fundoplication is the short “floppy” Nissen fundoplication, which has been shown to have success rates of up to 90% with minimal morbidity and mortality. 

Patients with Barrett's esophagus usually suffer from severe GERD, and antireflux operations offer potential advantages by restoring the LES pressure and abolishing gastric or alkaline reflux into the esophagus [129]. The effect of antireflux surgery on the natural history of Barrett's esophagus is a matter of controversy. Several studies document the efficiency of surgical therapy in the prevention of intestinal metaplasia in GERD patients[129, 130]. Others did not document any regression of intestinal metaplasia after antireflux procedures [131, 132]. Despite the fact that complete regression rarely occurs, regression of the length of Barrett's epithelium is commonly observed [130, 133]. Furthermore, disease progression, after antireflux operation, to severe dysplasia or adenocarcinoma occurs in a reduced incidence compared with medical therapy [129]. This is explained by the fact that fundoplication creates a new antireflux valve, which prevents both acid and bile reflux, a prerequisite for the development of Barrett's esophagus [134].

#### 5.2.2. Laparoscopic Surgery

Over the last fifteen years the advent of laparoscopic surgery has changed the way in which antireflux surgery is performed, with the associated advantages of minimally invasive surgery, rendering esophageal wrapping more acceptable. Despite the fact that open surgery for GERD was reserved for patients with severe symptomatology or complications, the introduction of laparoscopy in the management of the disease has resulted in a trend to operate at an earlier stage [135]. The first series of laparoscopic fundoplication was first reported in 1991, by Geagea from Canada [136], and Dallemagne from Belgium [137]. Since then, this operation has spread all over the world and was introduced in the routine clinical practice as the preferred surgical treatment for GERD. The advantages of this technique are reduced pain, less surgical trauma, shorter hospital stay, and better cosmetic result. Several studies have reported excellent short term results for this procedure [114, 116, 138]. However, other reports have emphasized the high incidence of early postoperative complications, such as the paraesophageal migration of the wrap or the stomach [139, 140]. With increasing experience, mortality after laparoscopic Nissen fundoplication has been reported to be very limited, not exceeding 0.1%. However, patients have to get used to several side effects, such as inability to vomit or to belch, and/or mechanical obstruction of the swallowed bolus, due to the wrap construction [141]. Moreover, dysphagia of sufficient severity to require esophageal dilation takes place in about 6% of patients after antireflux surgery [142, 143]. A recent randomized clinical trial suggests that laparoscopic Nissen fundoplication is associated with more obstructive complaints in the early postoperative period than the open approach [142]. However, this has not been reconfirmed by other authors [144]. There is a consensus among laparoscopic surgeons that the Nissen flap has to be both short and floppy to avoid early dysphagia [141]. Regarding long-term clinical outcome, the laparoscopic approach offers good to excellent results after a follow-up period longer than 5 years; however, there is still a possibility of technical failure, and the reoperation rate varies between 4% and 13% [145]. Dallemagne et al. [146] reported that 90% of a group of patients had symptom control 10 years after surgery, and that less than 10% of patients had to resume medication again. They also observed more recurrences after partial posterior fundoplication (Toupet) than after Nissen fundoplication. On the other hand, recurrent reflux after a Nissen fundoplication may often require a redo operation because of the accompanying dysphagia. In cases with severe persistent dysphagia, Nissen fundoplication should be taken down and changed to a Toupet procedure [147, 148]. 

Persistent dysphagia due to a very tight wrap, wrap disruption, incorrect placement and slippage of the wrap are causes of failure and require reoperation [149]. Early identification of these problems may urge to laparoscopic revision. However, later identification, beyond the first postoperative week, often requires more extensive open procedures. Experienced surgeons may perform revisions laparoscopically, even months after the initial procedure [150]. At revision, complete reduction of the previous wrap, repair of the hiatus and a further fundoplication is performed. Symptomatic improvement is observed in the majority of patients after revisional surgery for dysphagia. Laparoscopic revision is technically demanding but can produce satisfactory results similar to these of the initial operation [151].

Another issue is based on the observation that patients with Barrett's esophagus who have undergone a fundoplication, which has subsequently failed, are at increased risk of developing esophageal cancer during long-term follow up, due to recurrent reflux. For that reason if the fundoplication has failed, the patients should resume PPI medication until a laparoscopic revision is performed [134].

Laparoscopic fundoplication has replaced the open approach in most centers, being more acceptable by surgeons and patients. This approach can effectively control GERD symptoms and improve quality of life even in patients with recalcitrant GERD [151]. The laparoscopic approach has also been proven equally effective as the open approach in controlling GERD symptoms. Although the dysphagia rate after both procedures is similar, the open approach has a higher incidence of postoperative chest and wound complications. Moreover, the incidence of temporary gastric fullness and bloating syndrome is higher with the open approach [152]. In general, laparoscopic fundoplication is an accurate procedure with an acceptable complication rate and easily accessible to the general surgeon. Physicians and surgeons are now not reluctant to refer for or to perform laparoscopic fundoplication at least in selected patients.

#### 5.2.3. Endoluminal Surgery

The last few years novel endoscopic techniques have been introduced for the treatment of GERD. Endoluminal gastroplication (ELGP) was the first of the proposed endoscopic treatments for GERD. EndoCinch (C.R. Bard Inc., Cranston, RI), a commercially available suturing system has been used for ELGP. The effectiveness of this system has been proved in multicenter trials in the West [153], and also in Japan [154]. The procedure was found to be safe and was effective in about 60% of patients with GERD [155]. However, it failed to normalize acid reflux, had some serious complications, and long-term durability data are lacking [156]. Another technique using the Plicator device (NDO Surgical, Inc., Mansfield, MA) has been widely used. This technique mimics the effects of antireflux surgery by recreating the antireflux barrier, restoring the angle of Hiss and forming an one-way gastroesophageal valve. The Plicator procedure has been tried in several centers and has been shown to reduce GERD symptoms and medication use for at least 36 months following initial treatment [155, 157]. Moreover, this procedure is free of major complications and generally well tolerated [157].

A more practical technique, the novel endoluminal fundoplication (ELF) technique has been introduced to overcome some of the Plicator's disadvantages, such as the inability to reduce hiatal hernia and create a robust gastroesophageal valve. According to the ELF technique, the gastroesophageal valve is created from the inside of the stomach via transoral access. The EsophyxX^TM^ device (Endogastric Solutions, Inc., Redmond, WA, USA) is introduced trasnorally to create a full thickness omega shaped valve 3–5 cm in length and 200–300° in circumference through delivery of multiple fasteners under endoscopic visualization [158]. This new technique results in the creation of robust and durable gastroesophageal valve, which improves the functionality of the antireflux barrier. A multicenter study is underway in Europe to assess the long-term efficacy of the ELF procedure [159].

### 5.3. Medical versus Surgical Treatment for GERD

Surgical treatment for GERD has previously been limited to cases with chronic complicated reflux and severe symptomatology not responding to medication. Today there is increased tendency worldwide to utilize surgery in the earlier stages of the disease [160]. This change in clinical practice is mainly due to advancements in surgical technique, the increased patient satisfaction by laparoscopy, and the increased awareness of the impairment in quality of life of patients who are not efficiently treated [161]. Moreover, the increasing enthusiasm of patients and surgeons for minimally invasive surgery has led to the wider application of laparoscopy in the management of GERD in many institutes worldwide. Although modern drug therapy is very effective in the long-term management of GERD, antireflux surgery seems to be more cost effective than medical therapy and safer regarding long-term effects of acid suppression and development of adenocarcinoma of the esophagus in patients with severe forms of the disease [162]. 

There are several trials favoring the clinical outcome of laparoscopic antireflux surgery compared to long-term PPI therapy. A large randomized clinical trial from the UK has shown significantly better physiological control of reflux in patients having undergone laparoscopic Nissen fundoplication than patients under maintenance PPI therapy [163]. There was also better general well being in the surgery group after a followup of 12 months. Nevertheless, a recent prospective study on laparoscopic Nissen fundoplication from J. Hunter's group from Atlanta [164] with an 11-year mean follow up, demonstrated a significant symptom control, increased patient satisfaction, and complete withdrawal of antireflux medications by 70% of patients. This represents strong evidence regarding the efficacy and durability of the laparoscopic approach. In addition, a recent prospective trial from the UK [165] comparing laparoscopic Nissen fundoplication with PPI therapy, with 7-year follow-up, demonstrated that all patients, no matter the type of therapy, had a significant symptom improvement after the initial 12 months; however, patients who underwent surgery despite having had optimal PPI treatment had further symptomatic improvement at long-term follow-up.

However, other authors report modest results after laparoscopic antireflux surgery. Balsara et al. [166] from India analyzed retrospectively their experience with laparoscopic fundoplication over an 8-year period. This operation was performed in 84 patients, and 74 of them had been followed up for a period of 7 months to 8 years. Of these patients 57 (77%) have had a good result from surgery. Seventeen (23%) had a poor result, due to wrap failures, delayed gastric emptying or gas bloating. The authors found individual variables predicting a good response to surgery, including a good response to PPIs, volume reflux, and a pH score of more than 14. They concluded that strict selection criteria are necessary to optimize the results of surgery. Poor selection may result in a patient who may not benefit from surgery, but may be worse than before surgery.

In addition other studies challenge the superiority of antireflux surgery in the treatment of esophagitis. Thus, Lundell et al. [167] have reported 5- and 7-year results of a randomized controlled trial of patients with esophagitis treated with omeprazole or surgery. At 7 years the two treatments were similar regarding the incidence of recurrent esophagitis (10.3% omeprazole versus 11.8% antireflux surgery). In addition the two therapies appeared to be equivalent in healing esophageal mucosa. 

Conflicting evidence also exists regarding the efficacy of antireflux surgery in case of Barrett's esophagus. Previous reports support laparoscopic antireflux surgery as the most effective treatment for Barrett's esophagus, as it provides a durable reflux barrier, stimulates regression of intestinal metaplasia, and reduces the risk of adenocarcinoma [168]. However, a recent meta-analysis [169] fails to prove any protective effect of surgery against esophageal cancer.

Although the superiority of surgery to PPI therapy regarding the symptomatic control of reflux is well recognized, there are no controlled data comparing the two treatments with respect to the extraesophageal syndromes. However, observational studies show some benefit with surgical treatment for highly selected patients with reflux cough syndrome [170] and reflux asthma syndrome [171].

As for the recently evolving endoluminal therapy, there are no studies comparing the efficacy of these devises with either medical therapy of antireflux surgery. According to some authors the plicator procedure could be a treatment option for patients with mild symptomatic GERD who do not wish to remain on PPIs and who do not currently wish to undergo surgical therapy [157]. However, due to the small numbers and the short follow up of these preliminary studies on endoluminal therapy in GERD [172], it is too early to draw conclusions for its efficacy and long-term outcome.

## 6. Conlusions

GERD is a very common disease with a broad spectrum of clinical symptoms and disorders. From a therapeutic perspective, GERD results from reflux of gastric contents into esophagus due to mechanisms including TLESRs and hiatal hernia. For diagnosis, PPI test is advocated as a simple diagnostic tool to identify patients with GERD. In cases with high likelihood of GERD and a negative PPI test, the best method to diagnose reflux is the 24-hour pH test alone or in combination with impedance.

PPIs are considered the best therapeutic option for the initial treatment of GERD patients. Symptomatic relapse is very frequent; therefore, most patients need a long-term treatment. The goals of an effective maintenance therapy are control of symptoms and prevention of complication. Medical and surgical treatments options are both effective as maintenance therapies. PPIs are the mainstay of medical long-term treatment. In patients with severe esophagitis, continuous PPI therapy with the lowest effective code based on symptom control is the appropriate long-term strategy. On-demand therapy is the reasonable strategy in the long-term management of patients with NERD.

Choosing the right candidates for surgery still remains a problem. Usually young patients who are willing to get rid of long-term maintenance medication are the best candidates for surgery. Also patients who are refractory to medical treatment, especially those with nocturnal regurgitation, and young patients with recurrent peptic strictures may benefit from surgery. Another important factor in determining the outcome of antireflux surgery is the surgeon's experience. It has been shown that low volume centers yield much poorer outcomes.

 The choice of the treatment schedule should always be individualized for every patient. It is therefore up to the patient, the physician and the surgeon to decide which the best treatment option is. Medical and surgical therapies for the GERD are not competing or they are not even complementary. They are probably the two sides of the same coin.
